# Rare Combined Small-Cell Lung Carcinoma With Lymph Node Metastasis Exclusive to the Small-Cell Component: A Case Report

**DOI:** 10.7759/cureus.94905

**Published:** 2025-10-19

**Authors:** Sara Bendadi, Oussama El Karnighi, Meryem El Ouazzani, Hanane Rais, Rhizlane Belbaraka

**Affiliations:** 1 Medical Oncology, Mohammed VI University Hospital of Marrakech, Marrakech, MAR; 2 Pathology, Mohammed VI University Hospital of Marrakech, Marrakech, MAR; 3 Anatomical Pathology, Mohammed VI University Hospital of Marrakech, Marrakech, MAR

**Keywords:** combined pulmonary carcinoma (cpc), combined small-cell lung carcinoma (c-sclc), non-small cell lung carcinoma (nsclc), rare cpc, small-cell lung carcinoma (sclc)

## Abstract

Combined small-cell lung carcinoma (C-SCLC) is a rare histological subtype characterized by the coexistence of small-cell and non-small-cell components, posing diagnostic and therapeutic challenges due to its heterogeneity and aggressive course. We report the case of a 65-year-old heavy smoker who presented with dyspnea and cough. Imaging revealed a right lower lobe mass, and he underwent pneumonectomy. Pathology showed C-SCLC composed of 70% squamous cell carcinoma and 30% small-cell carcinoma (Ki-67: 60%), with vascular invasion, pleural and bone infiltration, and nodal metastases (pT3N1Mx). Early post-operative recurrence with pleural and osseous metastases was observed, and cisplatin-etoposide chemotherapy was initiated. This case illustrates the poor prognosis of C-SCLC, often undetected on small biopsies due to sampling limitations, and underscores the importance of surgical specimens for accurate diagnosis. Given the absence of standardized treatment protocols, management requires a multidisciplinary and individualized approach.

## Introduction

Combined small-cell lung carcinoma (C-SCLC) is a rare histological subtype characterized by the coexistence of small-cell and non-small-cell components. It represents a diagnostic and therapeutic challenge due to its biological heterogeneity and aggressive behavior. Combined pulmonary carcinoma (CPC) may arise de novo (synchronously) or by transformation, often post-therapeutic [1]. SCLC, in general, is the most aggressive of the major lung cancer types, with the worst long-term prognosis and survival rates.

## Case presentation

**Figure 1:**
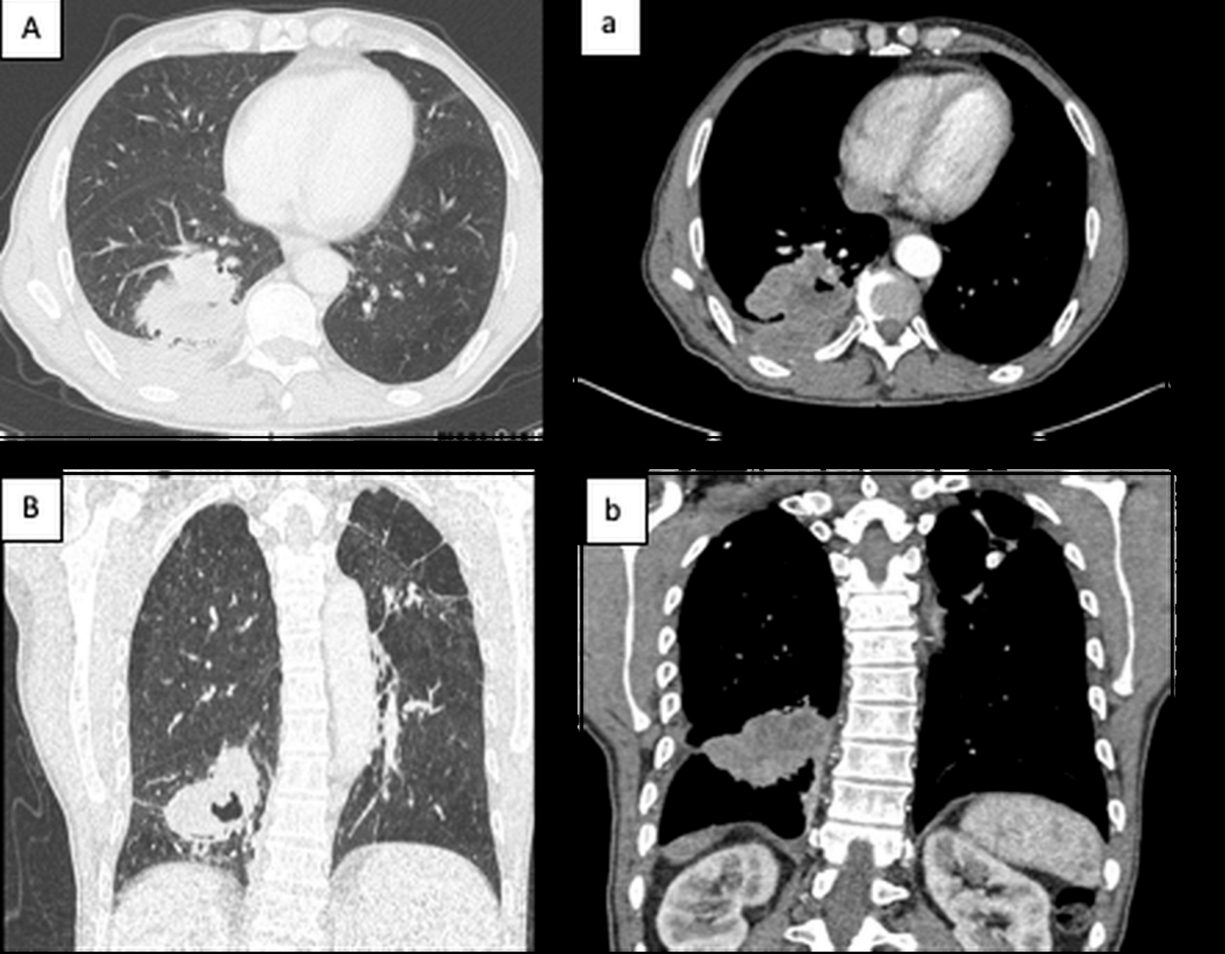
CT scan in coronal and axial parenchymal windows of the lesion process. Axial CT scan in parenchymal window (A) and mediastinal window (a) showing a right Fowler's lobe mass with cavitation and heterogeneous enhancement after contrast administration, coronal CT scan in parenchymal window (B) and mediastinal window (b) showing a right Fowler's lobe mass with cavitation and heterogeneous enhancement after contrast administration.

We report the case of a 65-year-old male with a significant smoking history of approximately 40 pack-years. He was referred to the University Hospital of Marrakech due to a deterioration in his general condition. His medical history dates back approximately four months, marked by the onset of exertional dyspnea and a productive cough, which was initially overlooked by the patient, leading him to consult a family doctor. He did not seek evaluation until May 2025. A CT scan showed a right Fowler's lesion measuring 53x50x58 mm, heterogeneously enhanced after product of contrast (PDC) injection. No mediastinal adenopathy (ADP) (Figure 1).

An initial tumor biopsy was not performed; instead, the patient proceeded directly to surgery based solely on the CT scan findings. He underwent surgery in March 2025 in the onco-thoracic surgery department. The main surgical specimen from the pneumonectomy corresponded to a combined small-cell lung carcinoma composed of two distinct cellular populations. The predominant component (approximately 70%) consisted of large polygonal tumor cells arranged in cohesive sheets, with atypical nuclei and abundant eosinophilic dyskeratotic cytoplasm, with occasional keratin pearls, consistent with moderately differentiated squamous cell carcinoma. The second component (approximately 30%) was composed of small tumor cells with hyperchromatic nuclei and scant eosinophilic cytoplasm, consistent with small-cell carcinoma.

The tumor measured 6.5 cm in greatest dimension and demonstrated vascular tumor emboli, with infiltration of the parietal pleura and adjacent osseous structures.

**Figure 2:**
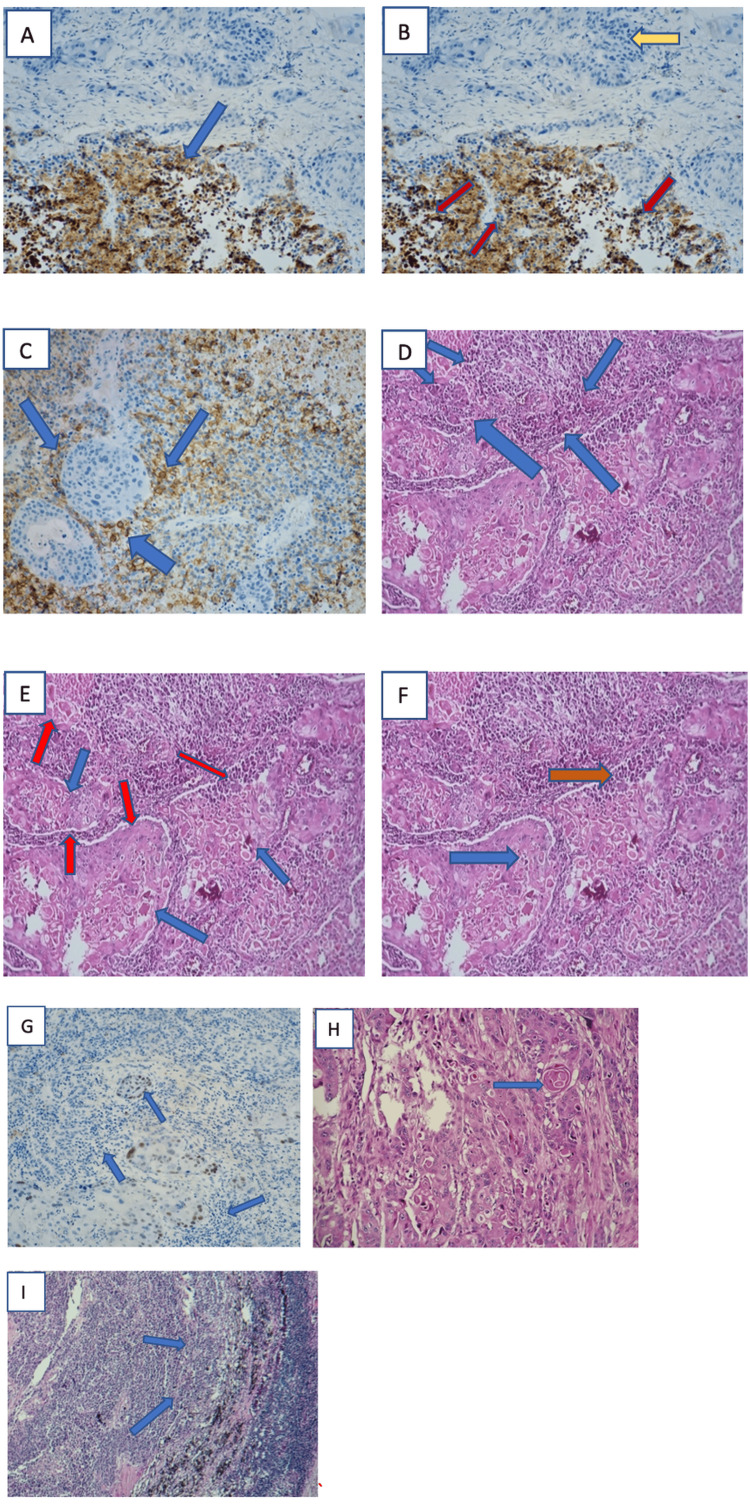
Morphological and pathological aspects of different components of the combined CPC. (A) Small-cell component showing granular cytoplasmic immunoreactivity for synaptophysin (Gx 20, blue (C) arrow). (B) The brown-stained nuclei indicate Ki-67 positive cells, reflecting high proliferative activity in the tumor area (bottom, red arrows). In contrast, the blue nuclei (hematoxylin counterstain) represent non-proliferating cells (Ki-67 negative, yellow arrows). The fibrous region at the top shows minimal staining, consistent with non-tumoral stroma. (C) Small-cell component showing membranous immunoreactivity for CD 56 (Gx 20, blue arrows). (D) Hyperchromatic nuclei and scant eosinophilic cytoplasm of small-cell carcinoma representing 30% of both components (neuroendocrine tumor, blue arrows). (E) Atypical nuclei (blue arrows) and abundant eosinophilic dyskeratotic cytoplasm (red arrows) of epidermoid carcinoma representing 70% of both components. (F) Biphasic tumor: moderately differentiated squamous cell carcinoma (blue arrow) and small-cell lung carcinoma (red arrow; H&E, ×10). (G) Squamous cell component showing immuno-positivity for P40 (Gx 20, blue arrows). (H) Moderately differentiated squamous cell carcinoma showing keratin pearls (blue arrow; H&E, Gx 20). (I) Lymph node metastasis of small-cell lung carcinoma (blue arrows; H&E, G× 10). CPC: combined pulmonary carcinoma.

Immunohistochemically, the squamous cell carcinoma component expressed P40, whereas the small-cell carcinoma component was positive for synaptophysin, chromogranin, CD56, and thyroid transcription factor-1 (TTF-1), with a high proliferative index (Ki-67 = 60%). Lymph node dissection revealed metastatic involvement of four lymph nodes by the small-cell carcinoma component, without evidence of capsular rupture. The hilar surgical margin was free of tumor infiltration. According to the TNM classification, 9th edition, the tumor was staged as pT3N1Mx (Figure 2).

**Figure 3:**
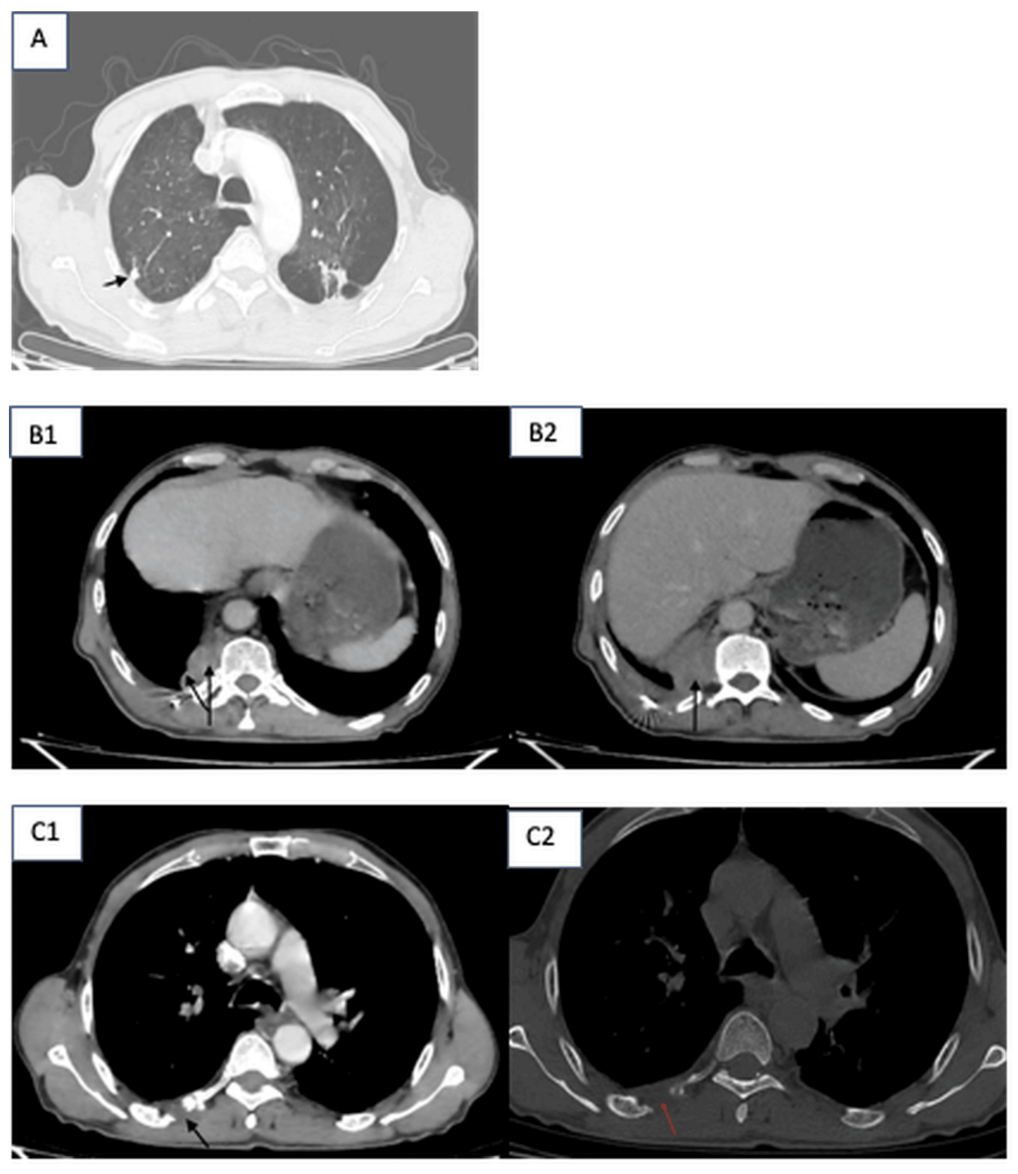
Post-operative CT scan in parenchymal, mediastinal, and bone windows. (A) Axial CT scan in parenchymal window showing a metastatic subpleural node measuring 15.9x16.5 mm. (B1) Axial CT scan in mediastinal window showing a right dorsobasal mass, with heterogeneous enhancement, and metastatic pleural nodes (B2), the largest measuring 13.9x19.5 mm. (C1) and (C2) Axial CT scan in bone and mediastinal windows showing metastasis involving the posterior arch of fifth right rib.

The patient was referred to the department of medical oncology. In May 2025, a post-operative CT scan was performed and showed a peripheral pleuropulmonary lesional process of the right Fowler and homolateral dorsobasal segment, locally infiltrating with two pleural nodules, one subpleural nodule, and lytic bone metastases involving the seventh and 11th ribs. All these findings had been discussed with the patient, and he was given the option to proceed. Chemotherapy with cisplatin and etoposide in combination with atezolizumab was initially planned; however, due to financial constraints, the patient could not receive atezolizumab (Figure 3). 

## Discussion

Combined small-cell lung carcinoma (C-SCLC) is a rare entity defined by the coexistence of both small-cell and non-small-cell lung cancer components within the same tumor [2,3]. In our case, the diagnosis was made postoperatively following a right lower lobectomy, highlighting the challenges of establishing this diagnosis on biopsy specimens alone. The diagnosis of C-SCLC is often difficult to make on biopsy alone, due to the heterogeneous nature of the tumors and the limited size of the specimens. This is why complete histological analysis on surgical specimens remains the gold standard for accurately identifying the various histological components [4].

The histogenesis, or origin, of these mixed tumors is not fully understood, but several theories exist. The most widely supported suggests divergent differentiation from a common progenitor cell, capable of giving rise to both SCLC and non-small cell lung carcinoma (NSCLC) components, as evidenced by the identical TP53 mutations observed in both subtypes [5]. Another hypothesis suggests transdifferentiation, whereby SCLC cells evolve into NSCLC cells via gene expression changes, notably around the Notch1 gene. Finally, the theory of a collision between two distinct tumors is less likely, given the genetic homogeneity frequently observed. It is likely that the origin of C-SCLC results from a combination of these mechanisms.

There is currently no established consensus on the optimal management of combined small-cell lung carcinoma (C-SCLC) [6]. In practice, treatment generally follows SCLC guidelines due to its aggressive nature [7]. While considering NSCLC-oriented strategies in selected cases, for example, (localized disease amenable to surgery or presence of actionable mutations) [8]. Thus, management should prioritize the most aggressive component, usually the SCLC.

Surgery remains a valuable option when resectability is possible, particularly in early stages [9]. However, systemic chemotherapy, often with platinum-based regimens, is essential due to the high risk of recurrence and metastasis. The role of immunotherapy in this setting is still under investigation, although emerging data from trials on extensive-stage SCLC may guide future approaches.

From a clinical standpoint, C-SCLC behaves more aggressively than pure NSCLC and may follow a course similar to that of SCLC, characterized by rapid progression and early metastasis. Reported median survival for patients with C-SCLC ranges between eight and 13 months, highlighting the poor prognosis associated with this entity. In our case, postoperative imaging revealed signs of pleural infiltration and bone lesions suggesting systemic disease [10].

This case underscores the importance of considering C-SCLC in patients with heterogeneous clinical or histological presentations. A multidisciplinary approach involving oncologists, pathologists, surgeons, and radiologists is crucial for accurate diagnosis and optimal management.

## Conclusions

Combined small-cell lung carcinoma (C-SCLC) remains a rare and underrecognized entity, whose diagnosis is often missed on initial biopsies due to its histological heterogeneity. Our case illustrates the diagnostic value of surgical specimens in identifying mixed histological components and the importance of considering C-SCLC in patients with atypical presentations or unexpected histological findings.

Given its aggressive nature and the absence of standardized therapeutic protocols, the management of C-SCLC requires a personalized and multidisciplinary approach. While surgery may offer diagnostic clarity and therapeutic benefit in localized cases, systemic treatment remains essential due to the high risk of recurrence and metastasis. It is recommended that if a tumor is composed of more than one histological pattern and is found to contain any proportion of SCLC cells, it should be classified as C-SCLC, regardless of which component is predominantly seen in the mass.
